# Genomic characterization and multidrug resistance of *Pseudomonas aeruginosa* isolated from peregrine falcons in Saudi Arabia: A One Health perspective

**DOI:** 10.14202/vetworld.2025.1964-1971

**Published:** 2025-07-22

**Authors:** Ali Wahdan, Mahmoud Mohamed, Mahmoud M. Elhaig, Mohammed Al-Rasheed, Ehab M. Abd-Allah

**Affiliations:** 1 Department of Bacteriology, Immunology, and Mycology, Faculty of Veterinary Medicine, Suez Canal University, Ismailia, Egypt; 2 Department of Clinical Sciences, College of Veterinary Medicine, King Faisal University, Al-Ahsa, Saudi Arabia; 3 Department of Animal Medicine (Infectious Diseases), Faculty of Veterinary Medicine, Suez Canal University, Ismailia, Egypt; 4 Veterinary Hospital, Faculty of Veterinary Medicine, Zagazig University, Zagazig, Egypt

**Keywords:** antimicrobial resistance, falcons, One Health, phylogenetics, *Pseudomonas aeruginosa*, whole-genome sequencing

## Abstract

**Background and Aim:**

*Pseudomonas aeruginosa* is a multidrug-resistant (MDR) zoonotic pathogen increasingly implicated in infections in both humans and animals, including avian species. Raptors, particularly peregrine falcons, are vulnerable due to their exposure to diverse environments and intensive management practices. This study aimed to identify *P. aeruginosa* isolates from peregrine falcons in Saudi Arabia and to characterize their genomic features, phylogenetic relationships, and antimicrobial resistance (AMR) profiles using whole-genome sequencing (WGS).

**Materials and Methods:**

Eighty cloacal swabs were collected from adult peregrine falcons showing clinical signs of gastrointestinal distress and housed in falconry facilities in Eastern Saudi Arabia between 2022 and 2024. Samples underwent bacteriological culture, biochemical identification using the Vitek 2 system, and WGS of a representative isolate. Multilocus sequence typing (MLST) analysis, phylogenetic comparison, and resistance gene profiling were conducted using standard bioinformatic tools and the Comprehensive Antibiotic Resistance Database and PubMLST databases.

**Results:**

Sixteen isolates (20%) were confirmed as *P. aeruginosa* through biochemical identification and BLAST analysis. One representative isolate underwent WGS and revealed a 6.0 Mbp genome with close phylogenetic relatedness (92% bootstrap) to a human-derived *P. aeruginosa* strain (CP050326), with a 4% genetic divergence. The MLST profile included allele numbers *acsA* (23), *aroE* (29), *guaA* (1), *mutL* (3), *nuoD* (1), *ppsA* (15), and *trpE* (9). Resistance genes identified included β-lactamase PAO-type (*blaPAO*), Class Dβ -lactamase OXA-type (variants 485 and 488) (*blaOXA-485/488*), aminoglycoside 3′-phosphotransferase type IIb (*aph(3′)-IIb*), glutathione transferase FosA (*fosA*), and chloramphenicol acetyltransferase type B7 (*catB7*), indicating MDR to beta-lactams, aminoglycosides, fosfomycin, and chloramphenicol.

**Conclusion:**

This is the first report of WGS-characterized, MDR *P. aeruginosa* in falcons from Saudi Arabia. The genomic similarity to human strains highlights the zoonotic potential and One Health implications. These findings emphasize the urgent need for integrated AMR surveillance in wildlife, especially in regions with widespread falconry practices. WGS offers valuable molecular insights for pathogen tracking, resistance monitoring, and epidemiological risk assessment. Broader genomic surveillance across bird species and regions is necessary to guide control strategies and reduce the risks of zoonotic transmission.

## INTRODUCTION

Avian behavior and migratory patterns play a pivotal role in shaping the geographical distribution of infectious agents, serving as key epidemiological determinants for several pathogens [1]. Among these, *Pseudomonas aeruginosa* has emerged as a notable opportunistic pathogen in raptor populations, capable of causing a range of infections under conditions of immunosuppression or environmental stress [2].

*P. aeruginosa* is an aerobic, Gram-negative, rod-shaped bacterium widely recognized for its oppo-rtunistic pathogenicity. The clinical management of infections caused by this organism is complicated by its intrinsic resistance mechanisms and remarkable ability to acquire additional resistance traits [3]. The misuse and overuse of antibiotics in veterinary settings and livestock production have further accelerated the emergence and dissemination of antimicrobial-resistant strains [4]. Given its frequent isolation from both human and animal hosts, *P. aeruginosa* is classified as a zoonotic pathogen. Report by Badawy *et al*. [5] has documented multidrug-resistant (MDR) strains in various sour-ces, including birds, livestock, environmental niches (e.g., bedding, water, and feed), and even on the hands of veterinary staff and animal handlers.

Modern molecular epidemiology has increasin-gly relied on multilocus sequence typing (MLST) for accurate characterization of bacterial strains across surveillance networks. Within the European Union and the European Economic Area, core genome MLST (cgMLST) has become the gold standard for high-resolution genotyping of bacterial pathogens [6]. The advent of affordable whole-genome sequencing (WGS) technologies has enabled comprehensive and detailed differentiation of bacterial isolates. However, effective utilization of WGS data requires advanced bioinformatics expertise and robust analytical platforms [7].

Globally, *P. aeruginosa* is a major contributor to MDR infections. Its resistance profile is enhanced by a combination of horizontal gene transfer and adaptive mechanisms, allowing resistance to multiple antibiotic classes [8]. As a member of the ESKAPE group of pathogens, *P. aeruginosa* presents unique therape-utic challenges due to its complex regulatory networks that mediate antimicrobial resistance (AMR) and persistence [9].

Although polymerase chain reaction (PCR)-based techniques have traditionally been employed to detect resistance genes, these methods are limited by their reliance on known targets and cannot detect novel or unexpected resistance determinants [10]. In contrast, high-throughput WGS, combined with bioinformatics pipelines, offers a powerful tool for the rapid and comprehensive identification of resistance genes in bacterial genomes [11].

Despite increasing reports of *P. aeruginosa* in both clinical and environmental settings, there is a paucity of genomic data on MDR strains isolated from avian wildlife, particularly raptors such as peregrine falcons. Most available studies rely on phenotypic identification or PCR-based detection of known resistance genes, which may underestimate the full spectrum of resistance determinants. Furthermore, there is limited information on the phylogenetic relatedness between avian-derived *P. aeruginosa* strains and those from human or environmental sources, hindering our understanding of potential cross-species transmission and One Health implications. To date, no WGS-based investigation has been conducted on *P. aeruginosa* isolated from falcons in Saudi Arabia, a region with active falconry practices and increased exposure risks.

This study aimed to isolate and identify *P. aeruginosa* from peregrine falcons in Saudi Arabia and to characterize their AMR profiles and phylogenetic relationships using WGS. By integrating MLST, resistance gene annotation, and phylogenetic analysis, this work sought to provide a comprehensive genomic overview of avian *P. aeruginosa* strains and assess their potential risk to animal and public health within a One Health framework.

## MATERIALS AND METHODS

### Ethical approval

All animal-related procedures were performed following institutional ethical guidelines and were approved by the Scientific Research Ethics Committee of the Faculty of Veterinary Medicine, Suez Canal University (Approval No. SCU-Vet-AREC2025003).

### Study period and location

The study was conducted from December 2022 to December 2024. Cloacal swabs were collected from 80 adult peregrine falcons (aged >2 years) housed in falconry facilities located in the Eastern Province of Saudi Arabia.

### Sampling and study design

Birds presenting with signs of gastrointestinal distress, such as diarrhea and lethargy, at the Avian Clinic, Veterinary Teaching Hospital, King Faisal University, were selected for sampling. The cloacal swabs were inoculated into Tryptic Soy Broth and incubated at 37°C for 24 h. The study included bacteriological isolation, biochemical identification, genetic characterization, and AMR profiling of *P. aeruginosa* isolates.

### Bacterial isolation and biochemical identification

Following incubation, enriched samples were streaked onto Tryptic Soy Agar, MacConkey Agar, and Cetrimide Agar (Himedia, Mumbai, India) for the selective isolation of *P. aeruginosa*. Distinct colonies were sub-cultured and identified based on morphological features and pigment production. Biochemical identification was performed using the Vitek 2 Compact system (BioMérieux, France), adhering to the manufacturer’s guidelines. As described by Elhaig *et al*. [12], Gram-negative isolates were suspended in Vitek 2 saline to achieve a turbidity of 0.52–0.62, as measured by DensiCHEK Plus (BioMérieux, France). The bacterial suspensions were then loaded into the Vitek 2 system using Gram-negative identification cards, and biochemical activity profiles were analyzed.

### WGS

One phenotypically representative *P. aeruginosa* isolate was selected for WGS. Genomic DNA was extracted using the PureLink Genomic DNA Kit (Invitrogen, CA, USA) and quantified with a Qubit Fluorometer using the High-Sensitivity DNA Assay Kit (Thermo Fisher, Eugene, USA). DNA libraries were constructed using the Illumina Nextera XT Kit and sequenced using a MiSeq platform with 2 × 250 bp paired-end reads (500-cycle v2 chemistry), as described by Ma *et al*. [13]. Raw reads were quality-checked using FastQC [14], and low-quality sequences and adapters (Phred score < Q30) were trimmed using Cutadapt v1.18 [15].

### MLST

MLST analysis was carried out using seven housekeeping genes: *acsA, aroE, guaA, mutL, nuoD, ppsA*, and *trpE*, based on the established *P. aeruginosa* MLST scheme [16]. Sequence alignment was performed using Clustal v2.1, and trimming was done with Mesquite v3.40 [17]. Phylogenetic trees were constructed using IQ-Tree v1.6.10 with 1,000 ultrafast bootstrap replicates, employing the best-fit substitution models selected through the Akaike Information Criterion [18]. Pairwise genetic distances (p-distance) were calculated using PAUP* v.4.0a platform through the CIPRES Science Gateway [19].

### Allelic profiling

Allelic profiles were determined by querying the PubMLST *P. aeruginosa* database (https://pubmlst.org/paeruginosa). Alleles that differed by one or more base pairs from existing entries were classified as novel variants [20].

### Resistance gene annotation

AMR genes were identified by aligning assembled genomic sequences against the Comprehensive Antibiotic Resistance Database using BLASTn. Genes showing ≥90% sequence identity and ≥80% coverage were considered present and annotated accordingly.

## RESULTS

### Phenotypic identification of *P. aeruginosa*

All 16 recovered *P. aeruginosa* isolates appeared as Gram-negative rods, either singly or in short chains, under microscopic examination. On cetrimide agar (HiMedia, Mumbai, India), the colonies were large, asymmetrical, and emitted a fruity odor, accompanied by the production of a characteristic yellowish-green fluorescent pigment. On MacConkey agar, the colonies were flat, smooth, and pale in color, consistent with non-lactose-fermenting bacteria. Biochemical identification using the VITEK 2 system (BioMérieux, France) confirmed that all 16 isolates were *P. aeruginosa*. This identification was further validated through BLAST analysis, which revealed a 92% identity match with the *P. aeruginosa* genome sequence available in GenBank.

### WGS and phylogenetic analysis

One representative *P. aeruginosa* isolate was selected for WGS using the Illumina MiSeq platform. The resulting genome assembly was 6,037,891 base pairs in length and corresponded to a reference *P. aeruginosa* strain (BioSample Accession: SAMN46967384), harboring six confirmed AMR genes.

**Figure 1:**
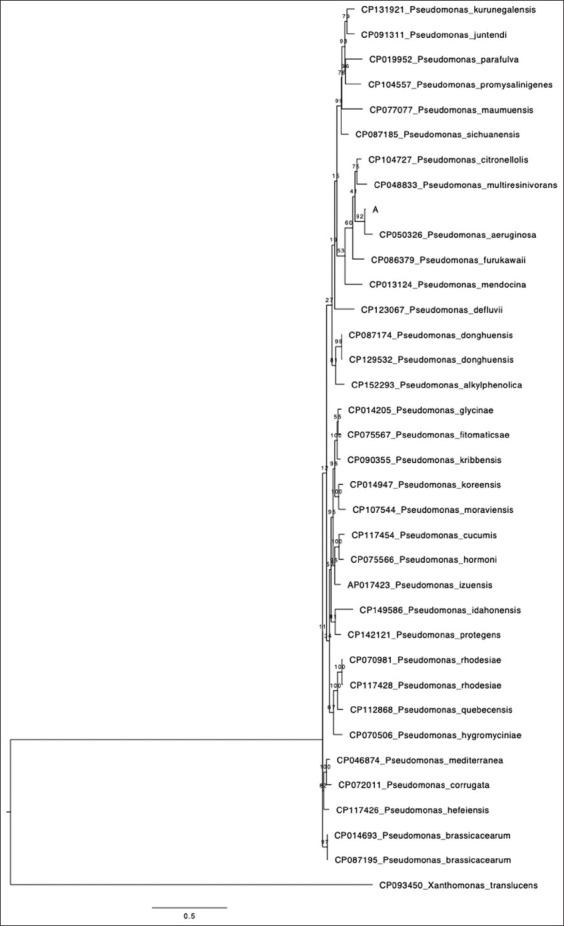
Phylogenetic tree of Saudi *Pseudomonas aeruginosa* and reference *Pseudomonas* species. The *P. aeruginosa* falcon strain characterized in this study is indicated as “A.”

Maximum likelihood phylogenetic analysis of the isolate, along with 30 reference Pseudomonas sequences, revealed that the tested isolate clustered closely with a human-derived *P. aeruginosa* strain (Accession No. CP050326, isolated from lung tissue, USA). This clustering was supported by a high bootstrap value of 92% and a genetic divergence (p-distance) of only 4%. The phylogenetic tree (Figure 1) demonstrated considerable genetic diversity among *Pseudomonas* isolates derived from varied sources such as falcons (this study), soil, wastewater, plants, humans, antibiotic containers, and animal tissues and across numerous countries including Saudi Arabia, USA, Canada, Argentina, China, Japan, South Korea, Belarus, Denmark, Spain, Germany, Cameroon, and Nigeria.

### MLST allelic profile

MLST analysis of the isolate revealed the following allelic profile: *acsA* (23), *aroE* (29), *guaA* (1), *mutL* (3), *nuoD* (1), *ppsA* (15), and *trpE* (9). These results are summarized in Table 1 and confirm the genetic identity and classification of the isolate within the *P. aeruginosa* population structure.

**Table 1:** Allele lengths and variable sites of MLST fragments.

Locus	Importance	Allele length	Allele variable site
*acsA*	Acetyl coenzyme A synthetase	390	23
*aroE*	Shikimate dehydrogenase	498	29
*guaA*	GMP synthase	373	1
*mutL*	DNA mismatch repair protein	442	3
*nuoD*	NADH dehydrogenase I chains C and D	366	1
*ppsA*	Phosphoenolpyruvate synthase	370	15
*trpE*	Anthranilate synthase component I	443	9

MLST=Multilocus sequence typing

### Antibiotic resistance profile

Genomic analysis identified five antibiotic resis-tance genes with nucleotide identity ranging from 90.75% to 99.22% (Table 2). Based on the presence of these genes, the isolate was classified as a MDR strain. Resistance genes included beta-lactamase genes conferring resistance to ampicillin, amoxicillin, cefepime, ceftazidime, and an unidentified beta-lactam compound. In addition, genes indicating resistance to an uncharacterized aminoglycoside, chloramphenicol, and fosfomycin were also detected. These findings highlight the extensive resistance potential of the *P. aeruginosa* strain isolated from peregrine falcons.

**Table 2:** Results of the WGS-resistant *Pseudomonas aeruginosa* strain.

Resistance gene	Class	Phenotype	Identity (%)	Length of alignment (bp)	Position in reference	Position in contiguous	Reference accession number
*blaPAO*	Beta-lactam	Amoxicillin	99.75	1194	1–1194	659 - 1852	AY083592/ AY083595
		Ampicillin					
		Cefepime					
		Ceftazidime					
*aph(3’)-IIb*	Aminoglycoside	Unknown aminoglycoside	99.50	807	1–807	14210 - 15016	CP006832
*blaOXA-485/* *blaOXA-488*	Beta-lactam	Unknown beta-lactam	99.37	789	1–789	18514 - 19302	LLNM01000004/CP017969
*fosA*	Fosfomycin	Fosfomycin	99.26	408	1–408	6842 - 7249	ACWU01000146
*catB7*	Amphenicol	Chloramphenicol	99.22	639	1–639	9524 - 10162	AF036933

WGS=Whole-genome sequencing

## DISCUSSION

### Susceptibility of falcons to *P. aeruginosa*

Falcons are susceptible to a wide range of opportunistic infections, including those caused by *P. aeruginosa*. The pathogen’s ability to form biofilms and exhibit MDR complicates clinical management and therapeutic outcomes. In the current study, *P. aeruginosa* was identified in 16 falcons, a finding that contrasts with earlier reports questioning the bacterium’s pathogenic role in falcons [21]. However, these results align with the findings of Naldo and Samour [22], who reported *P. aeruginosa* as one of the most prevalent bacterial infections among falcons in Saudi Arabia. While both Saker and Peregrine falcons were affected, no significant difference in infection rates was observed between species.

According to a previous study by Muller *et al*. [21], the majority of infected falcons were 1-year old (61.0%), followed by those aged 2 years (21.9%), 3 years (12.2%), and 4 years (4.9%). Increased susceptibility in falcons is often associated with immunosuppression due to stress, malnutrition, or overcrowding. In addition, underlying diseases or physical injuries may facilitate bacterial colonization and systemic dissemination [2].

### Diagnostic utility of the Vitek 2 system

The Vitek 2 Compact system demonstrated a high diagnostic accuracy in this study, correctly identifying *P. aeruginosa* with an efficiency of approximately 98%. This automated platform offers rapid identification and antimicrobial susceptibility profiling of bacterial isolates [23]. However, the system’s accuracy may be affected by the quality of the sample and the interpretation of the results. Limitations include high operational costs, the need for trained personnel, and potential misidentification of rare or atypical bacterial strains [24].

### Genomic characteristics of *P. aeruginosa*

WGS revealed that *P. aeruginosa* possesses a large genome, typically ranging from 5.5 Mbp to 7 Mbp [25]. The core genome is well-conserved and collinear across strains, with interclonal sequence divergence estimated to be between 0.5% and 0.7% [26]. However, the accessory genome exhibits high variability, largely due to horizontal gene transfer and genomic plasticity, with approximately 79 loci identified that introduce novel genetic elements and contribute to strain-specific traits [27,28].

Although WGS provides comprehensive insights into chromosomal mutations and resistance genes, it presents challenges in data interpretation and requires specialized bioinformatic expertise. Furthermore, resistance determinants located in non-coding or regulatory regions may remain undetected [7].

### Epidemiological significance of MLST and cgMLST

MLST allele profiling provides high-resolution discrimination of bacterial isolates, making it a valuable tool for epidemiological surveillance, outbreak detection, and tracking. cgMLST extends the principles of MLST to a broader set of conserved genes, enhancing resolution and standardizing allele assignment [27]. Commercially available platforms now support the implementation of cgMLST schemes, which have already proven effective in tracking several bacterial pathogens [20].

### Resistance profile and public health implications

Five resistance genes were identified in the *P. aeruginosa* genome with nucleotide identities ranging from 90.75% to 99.22% (Table 2). These included beta-lactamase genes conferring resistance to ampicillin, amoxicillin, cefepime, ceftazidime, and an unidentified beta-lactam. The isolate also showed resistance to an unknown aminoglycoside, chloramphenicol, and fosfomycin. This is consistent with findings by Zeng and Jin [29], who reported the aph(3′)-IIb gene in *P. aeruginosa*, encoding resistance to multiple aminoglycosides such as butirosin, kanamycin, neomycin, and seldomycin.

The innate resistance of *P. aeruginosa* to beta-lactams arises from chromosomal beta-lactamases, limited outer membrane permeability, and active efflux pump systems [8,30]. The increasing prevalence of MDR *P. aeruginosa* is a serious global health concern [25]. Effective countermeasures include the rational use of antimicrobials, stringent infection control practices, and the development of new antimicrobial agents [9]. According to the World Health Organization guidelines, proper hygiene, optimal nutrition, and biosecurity are crucial in preventing *P. aeruginosa* infections in falcons, particularly in the absence of an effective vaccine [31].

### Phylogenetic distribution and zoonotic potential

The phylogenetic analysis revealed a 4% genetic divergence between the falcon-derived *P. aeruginosa* isolate and other reference strains. The resulting tree (Figure 1) grouped sequences from multiple sources, including falcons (this study), humans, soil, wastewater, plants, and animal tissues. Isolates were derived from diverse countries such as Saudi Arabia, USA, Canada, Argentina, China, Japan, South Korea, Belarus, Denmark, Spain, Germany, Cameroon, and Nigeria.

This broad geographic and host distribution highlights the global dissemination and adaptability of *Pseudomonas* species. The observed genetic diversity, combined with the isolate’s MDR, underscores the urgent need for continued genomic surveillance. A previous retrospective study by Almaghrabi *et al*. [32] also found high genetic diversity among MDR *P. aeruginosa* isolates from hospital environments, reinforcing the need for novel antimicrobials and antibiofilm strategies.

### Zoonotic and One Health implications of AMR

The findings of this study underscore the importance of adopting a One Health perspective in addressing the spread of antimicrobial-resistant *P. aeruginosa*. Falcons, especially those housed in close proximity to humans in falconry settings, repre-sent a potential reservoir and transmission bridge for resistant pathogens. The genomic similarity observed between the falcon-derived isolate and human clini-cal strains highlights the likelihood of cross-species transmission, possibly facilitated by environmental contamination, shared handling equipment, or direct human-animal contact. Such interactions exemplify the interconnectedness of animal, human, and environ-mental health. Integrating wildlife surveillance into national AMR monitoring programs is therefore essential. This approach not only aids in the early detection of emerging resistance threats but also informs evidence-based interventions to protect both public and animal health. The incorporation of genomic tools, as demonstrated in this study, enhances the ability to trace the origin and evolution of resistance genes across sectors, supporting the development of unified policies in line with global One Health initiatives.

## CONCLUSION

This study presents the first WGS-based characterization of *P. aeruginosa* isolated from per-egrine falcons in Saudi Arabia. Of the 80 cloacal samples collected, 16 isolates (20%) were confirmed as *P. aeruginosa* through biochemical and molecular analyses. WGS of a representative isolate revealed a genome size of 6.03 Mbp and identified five AMR genes: *blaPAO, blaOXA-485/488, aph(3′)-IIb, catB7*, and *fosA*, which confer resistance to multiple antibiotic classes, including beta-lactams, aminoglycosides, chloramp-henicol, and fosfomycin. Phylogenetic analysis revealed a close genetic relationship (92% bootstrap support) between the falcon-derived isolate and a human lung isolate, suggesting possible interspecies transmission. MLST profiling further confirmed its distinct allelic identity.

The detection of MDR *P. aeruginosa* in falcons has significant implications for avian medicine, falconry management, and the control of zoonotic diseases. This highlights the need for targeted antimicrobial stewardship, routine bacterial surveillance in falconry settings, and enhanced biosecurity measures.

The integration of WGS, phylogenetic mapping, and resistance profiling provides a high-resolution epidemiological snapshot. The observed genetic similarity between avian and human isolates highlights the interconnectedness of animal, human, and environmental health, validating the One Health appr-oach in monitoring AMR.

Only one representative isolate underwent WGS, which limits the generalizability of resistance patterns and genetic diversity across the broader falcon population. In addition, the study did not assess phenotypic antimicrobial susceptibility or the capacity for biofilm formation.

Future research should include larger sample sizes across multiple falcon species and geographic regions, comparative genomics with environmental and human isolates, and longitudinal surveillance to assess AMR trends. Functional validation of resistance genes and the role of accessory genome elements in virulence should also be explored.

The emergence of MDR *P. aeruginosa* in falcons underscores the need for integrated genomic sur-veillance in wildlife populations. These findings provide valuable data for assessing AMR risk and reinforce the urgency of implementing collaborative One Health strategies to mitigate zoonotic transmission and preserve antimicrobial efficacy in both veterinary and human healthcare systems.

## AUTHORS’ CONTRIBUTIONS

AW: Conceptualized and designed the study. MM and MA: Collected samples and analyzed the data. MM, MA, and EMA: Performed the detection and antimicrobial sensitivity techniques. AW and MME: Analyzed the data, visualized, and drafted the manuscript. All authors have read and approved the final manuscript.
